# The oral vaccine intrOv is attenuated in infecting the lower airway and inducing chronic pathology

**DOI:** 10.1128/iai.00122-26

**Published:** 2026-04-15

**Authors:** Zhaoyang Liu, Caiting Li, Sydney Mahan, Jie Ren, Shuping Hou, Guangming Zhong

**Affiliations:** 1 Department of Dermatovenereology, Tianjin Medical University General Hospital/Tianjin Institute of Sexually Transmitted Disease 117865, Tianjin, China; 2 Department of Microbiology, Immunology, and Molecular Genetics, University of Texas Health Science Center at San Antonio 14742, San Antonio, Texas, USA; University of California Davis, Davis, California, USA

**Keywords:** IntrOv, airway attenuation, upper airway restriction

## Abstract

The *Chlamydia muridarum* mutant intrOv is an intracellular oral vaccine *v*ector, as oral inoculation with it induces protection in extra-gut mucosal tissues. Although intrOv is attenuated in both genital and gastrointestinal mucosal tissues, it remains unclear whether intrOv induces pathology in the airway, as wild-type *C. muridarum* causes severe lung pathology and body weight loss. The current study revealed that following intranasal inoculation, intrOv failed to induce significant weight loss and shed fewer live organisms than wild-type. Acute infection with intrOv was mainly restricted to the upper airway, with no live intrOv recovered from lung tissue, whereas the similarly inoculated wild-type disseminated throughout the airway. Despite the absence of live intrOv in the lung, intranasally inoculated intrOv still induced acute lung inflammatory infiltration, consistent with the detection of intrOv genomes in the lung. However, the intrOv-induced acute lung inflammation was fully resolved by day 28, whereas the wild-type-induced lung inflammation persisted with progression to fibrosis. Thus, intrOv is highly attenuated in both its infectivity and pathogenicity in the lower airway. Despite significant attenuation, the airway intrOv still induced chlamydial antigen-specific serum antibodies with a titer comparable to that induced by wild-type *C. muridarum*. Thus, we have demonstrated the airway safety of intrOv as an oral vaccine and provided evidence supporting its development as an airway vaccine to induce systemic immunity.

## INTRODUCTION

The medical burden caused by *Chlamydia trachomatis* is due to the chlamydial induction of pathological sequelae in the upper genital tract following sexually transmitted infection (1–4). Although *C. trachomatis* is susceptible to antibiotics, many infected individuals are not properly treated because specific symptoms are often absent. The most effective way to reduce the medical burden caused by *C. trachomatis* may be vaccination. However, there is still no licensed human vaccine to prevent *C. trachomatis* infection despite extensive efforts that began more than 70 years ago (5). The failure of the early formalin-fixed whole-organism-based trachoma vaccines motivated chlamydial researchers to develop subunit vaccines. Although some subunit vaccine candidates have advanced to preclinical testing (6) or clinical trials (7, 8), none have yet been licensed (9). Recent advances in understanding the mechanisms by which killed chlamydial organisms induce immune suppression have rekindled interest in developing a whole-cell-based chlamydia vaccine (10, 11).

The mouse-adapted chlamydial species, *C. muridarum*, has been used to investigate chlamydial interactions with host mucosal tissues and develop chlamydial vaccines (12). The *C. muridarum*-based studies revealed a complex relationship between chlamydia and host genital and gut mucosal tissues (13, 14). When a naïve mouse is first exposed to chlamydia in the gastrointestinal (GI) tract, GI chlamydia is not only non-pathogenic but can also function as an oral vaccine, inducing transmucosal protection against subsequent chlamydial infections in extra-gut mucosal tissues (14). Oral inoculation with *C. muridarum* mutants attenuated for genital pathogenicity also induced transmucosal protection in the genital tract (15, 16). Since *C. muridarum* shares a similar genome with *C. trachomatis* (17) and prior exposure to one chlamydial species confers cross-protection against the other species (18, 19), an attenuated *C. muridarum* clone, designated intrOv (20, 21), is being developed as a human vaccine for protection against *C. trachomatis* (22). IntrOv carries a substitution mutation in *tc0237*, resulting in the replacement of a Q residue at the 117 position with E (TC0237Q117E), and a nonsense mutation in *tc0668*, resulting in a premature stop codon for truncating the 408aa protein of TC0668 at G216 (TC0668G216*). These mutations may contribute to the intrOv’s attenuation in the mucosal tissues of the genital (21) and gastrointestinal (23) tracts. However, the precise mechanism of intrOv attenuation remains unknown.

Although intrOv is attenuated in pathogenicity in both the genital and gastrointestinal mucosal tissues, whether intrOv induces pathology in the airway remains unknown, even though wild-type *C. muridarum* (WtCm) causes severe lung pathology and body weight loss. The goal of the current study is to evaluate intrOv’s pathogenicity in the airway because it is important to ensure intrOv’s safety in the airway mucosal tissues, as there is always a chance for intrOv to contact the airway tissue during or after the administration of intrOv as an oral vaccine. We found that intranasal intrOv failed to induce significant body weight loss and shed significantly fewer live organisms than WtCm. Consistently, live intrOv infection was mainly restricted to the upper airway, with no live intrOv recovered from lung tissue, whereas WtCm disseminated throughout the entire airway. Although intrOv still induced acute lung inflammatory infiltration, the intrOv-induced pulmonary inflammation fully resolved by day 28, whereas the WtCm-induced lung inflammation persisted and progressed to fibrosis. Despite significant attenuation, intranasal intrOv still induced chlamydial antigen-specific serum antibodies with a titer comparable to that induced by WtCm. Thus, we have provided the first experimental evidence demonstrating attenuation of intrOv in the airway, thereby ensuring its safety as an oral vaccine. We have also provided information for potentially developing intrOv into a live-attenuated airway vaccine to induce systemic immunity.

## RESULTS

### The live-attenuated oral vaccine intrOv fails to induce systemic toxicity and sheds fewer live organisms following intranasal inoculation

**Fig 1:**
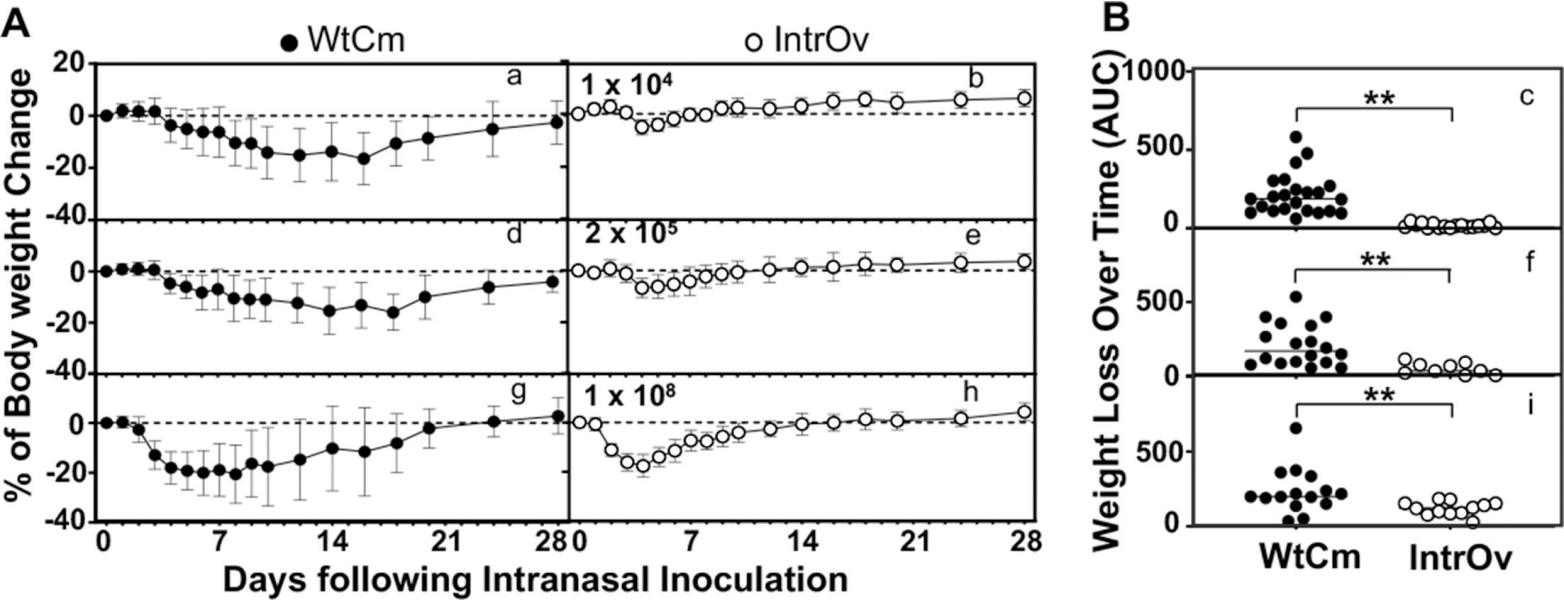
Longitudinal monitoring of mouse body weight following intranasal inoculation. Groups of C57BL/6J mice were intranasally inoculated with wild-type *Chlamydia muridarum* (WtCm) or the live-attenuated oral vaccine strain intrOv at different doses and weighed on the day prior to the inoculation (day 0) and then daily post-inoculation. (**A**) The results were expressed as % body weight change, displayed along the Y-axis over a 28-day time course as indicated along the X-axis following intranasal inoculation with WtCm (solid circles, left columns) at the dose of 1 × 10⁴ IFUs (panel a, *n* = 23), 2 × 10⁵ IFUs (d, *n* = 18), or 1 × 10⁸ IFUs (g, *n* = 15), or intrOv (open circles, right columns) at the dose of 1 × 10⁴ IFUs (b, *n* = 15), 2 × 10⁵ IFUs (e, *n*=9), or 1 × 10⁸ IFUs (h, *n* = 12). (**B**) The time-course weight loss was converted to AUC, as described in the Materials and Methods, and displayed on the Y-axis. The data were compiled from three to five independent experiments. The AUCs were compared between groups inoculated with WtCm or intrOv as indicated along the X-axis using two-tailed Wilcoxon. *P < 0.05 while **P < 0.01. Note that WtCm-induced body weight loss is significantly more severe than that following inoculation with intrOv, and the group sample sizes represent the number of mice at the beginning of each experiment. Some mice infected with WtCm died during the course of infection.

As one of the pathogenic hallmarks of *Chlamydia muridarum* infection in the mouse airway is body weight loss, we compared the weight changes over the course of 28 days following intranasal inoculation with different doses of wild-type *Chlamydia muridarum* (WtCm) or the live-attenuated oral vaccine intrOv (Fig. 1). We found that at a low dose of 1 × 10^4^ IFUs of WtCm, mice lost 10% or more body weight starting on day 8 post-inoculation, and this level of weight loss was maintained for up to day 18, and most mice were still unable to recover back to their original body weight prior to the intranasal inoculation. In contrast, mice similarly inoculated with intrOv only transiently lost about 5% of their body weight on day 4 after inoculation and then rapidly regained it. When mice were inoculated with a middle dose of 2 × 10^5^ IFUs of WtCm, 10% weight loss occurred on day 8, with a peak loss of ~17% on day 18, and the 10% of weight loss was extended to day 20. In comparison, the similarly inoculated intrOv only induced a maximal weight loss of ~7% on day 4, and mice quickly recovered their weight by day 10. Although WtCm induced 10% or more weight loss for 9 and 11 days at the low and middle inoculation doses, respectively, intrOv never induced 10% or more weight loss at any time point during the entire course of infection. However, when the inoculum was increased to a high dose of 1 × 10^8^ IFUs, both WtCm and intrOv induced significant weight loss, although they still differed in severity. WtCm induced >10% body weight loss as early as day 3 post-inoculation, and the loss was advanced to ~18% the next day and maintained for ~5 days. Only after an additional 4 days with a 10% loss did the WtCm-infected mice begin to regain body weight. At the same high inoculation dose with intrOv, the onset of 10% body weight loss was advanced to day 2, with a peak loss of ~18% on day 4. By day 7, the weight loss was reduced to <10%. Obviously, WtCm induced a more sustained weight loss (8 days) than intrOv (4 days). When the overall body weight losses were compared between the WtCm-infected and intrOv-infected mice, WtCm always induced more significant weight loss than intrOv, regardless of the inoculum doses used.

**Fig 2:**
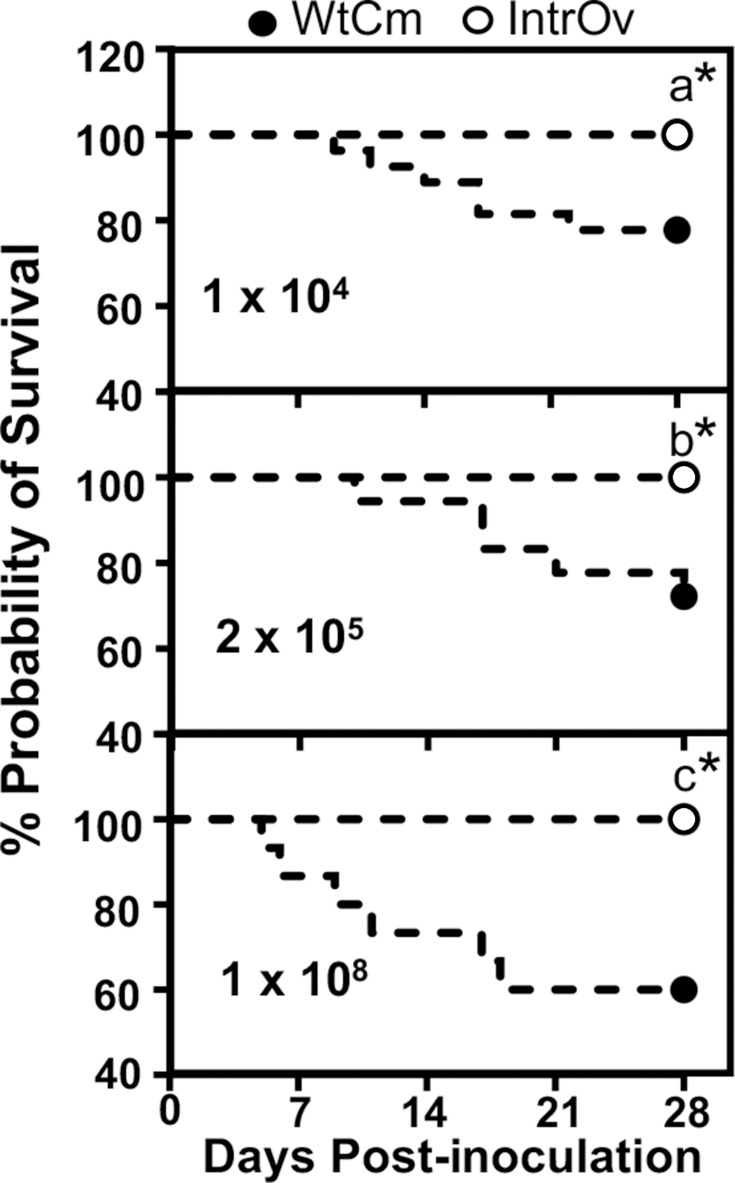
Survival of mice following intranasal inoculation with WT CM or intrOv. The same mice described in the legend of Fig. 1 were also monitored for survival. Following intranasal inoculation with WtCm (solid circle) or intrOv (open circle) at doses of 1 × 10⁴ IFUs (panel a), 2 × 10⁵ IFUs (b), or 1 × 10⁸ IFUs (c), mouse survival was monitored daily. Survival probabilities were plotted over time using Kaplan–Meier survival curves in Prism. Statistical significance between groups was determined using the log-rank (Mantel–Cox) test. *P < 0.05. Note that WtCm infection caused significant mortality, whereas none of the intrOv-inoculated animals died regardless of the inoculation doses.

Consistent with the more severe and sustained weight loss, WtCm also caused mouse mortality, whereas intrOv failed to do so (Fig. 2). At the low inoculation dose of 1 × 10⁴ IFUs with WtCm, 6 out of 23 mice died, while no death was observed in mice inoculated with intrOv. Similarly, both the middle and high doses of WtCm caused significant mortality, whereas no deaths occurred among intrOv-inoculated mice, regardless of the inoculation dose. IntrOv is likely 10,000-fold more attenuated in the airway than WtCm, as 1 × 10^4^ IFUs of WtCM caused significant mouse death, while 1 × 10^8^ IFUs of intrOv failed to do so.

**Fig 3:**
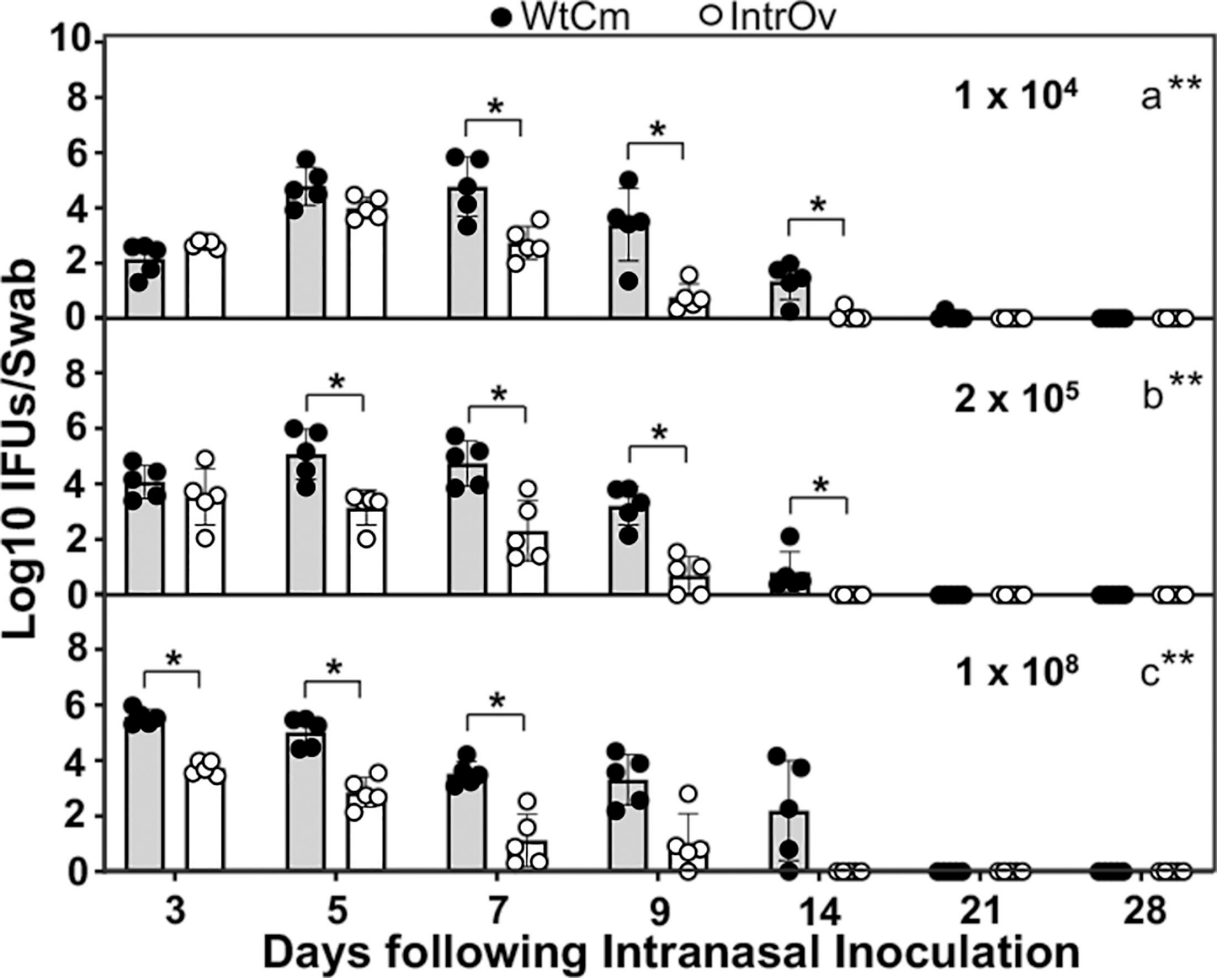
Oropharyngeal shedding of live organisms following intranasal inoculation with wild-type *Chlamydia muridarum* versus the attenuated vaccine strain intrOv. Groups of C57BL/6J mice (*n* = 5/group) were intranasally inoculated with WtCm (solid circles) or intrOv (open circles) at doses of 1 × 10⁴ IFUs (panel a), 2 × 10⁵ IFUs (b), or 1 × 10⁸ IFUs (c), as described in the Fig. 1 legend. On days 3, 5, 7, 9, 14, and weekly thereafter post-inoculation, oropharyngeal swabs were taken to titrate live chlamydial organisms as inclusion-forming units (IFUs). The results were expressed as Log_10_ IFUs per swab, shown along the Y-axis. The data were compiled from two independent experiments. Log_10_ IFUs detected at different time points following a given inoculation dose were compared between mice inoculated with WtCm or intrOv using ANOVA (all time points under a given inoculum dose) and Wilcoxon (at each time point). The time courses of live-organism shedding were converted to area-under-curves (AUCs) for comparison between the WtCm and intrOv groups, using ANOVA (all groups) and Wilcoxon (each dose), respectively. *P < 0.05, **P < 0.01, Wilcoxon (two-tailed). Note that both the shedding levels and time courses of intrOv were significantly lower and shorter than those of WtCm, regardless of the inoculation dose.

In parallel experiments, we also monitored the shedding of live chlamydial organisms in oropharyngeal swabs following intranasal inoculation (Fig. 3). At the low inoculum dose of 1 × 10^4^ IFUs, although WtCm and intrOv shed a similar level of live organisms on day 3, WtCm increased shedding significantly higher than intrOv by day 7. The WtCm shedding was consistently higher than the intrOv shedding for the remainder of the shedding course. When the inoculation dose was increased to the middle dose of 2 × 10^5^ IFUs, WtCm’s peak shedding was advanced to day 5, resulting in a highly significant difference in shedding level between WtCm and intrOv, which continued to day 14 post-inoculation. Interestingly, when the inoculation dose was increased to the high dose of 1 × 10^8^ IFUs, WtCm’s peak shedding was further advanced to day 3, resulting in a significant difference from intrOv throughout the entire shedding course. It is worth noting that as the inoculum dose increased from low to high, shedding peaks shifted from days 7 to 3 for WtCm and from days 5 to 3 for intrOv, respectively. When the overall shedding courses were compared, intrOv shedding was significantly lower than that of WtCm, regardless of inoculum dose.

### IntrOv is prevented from producing infectious organisms in the lung tissue

**Fig 4:**
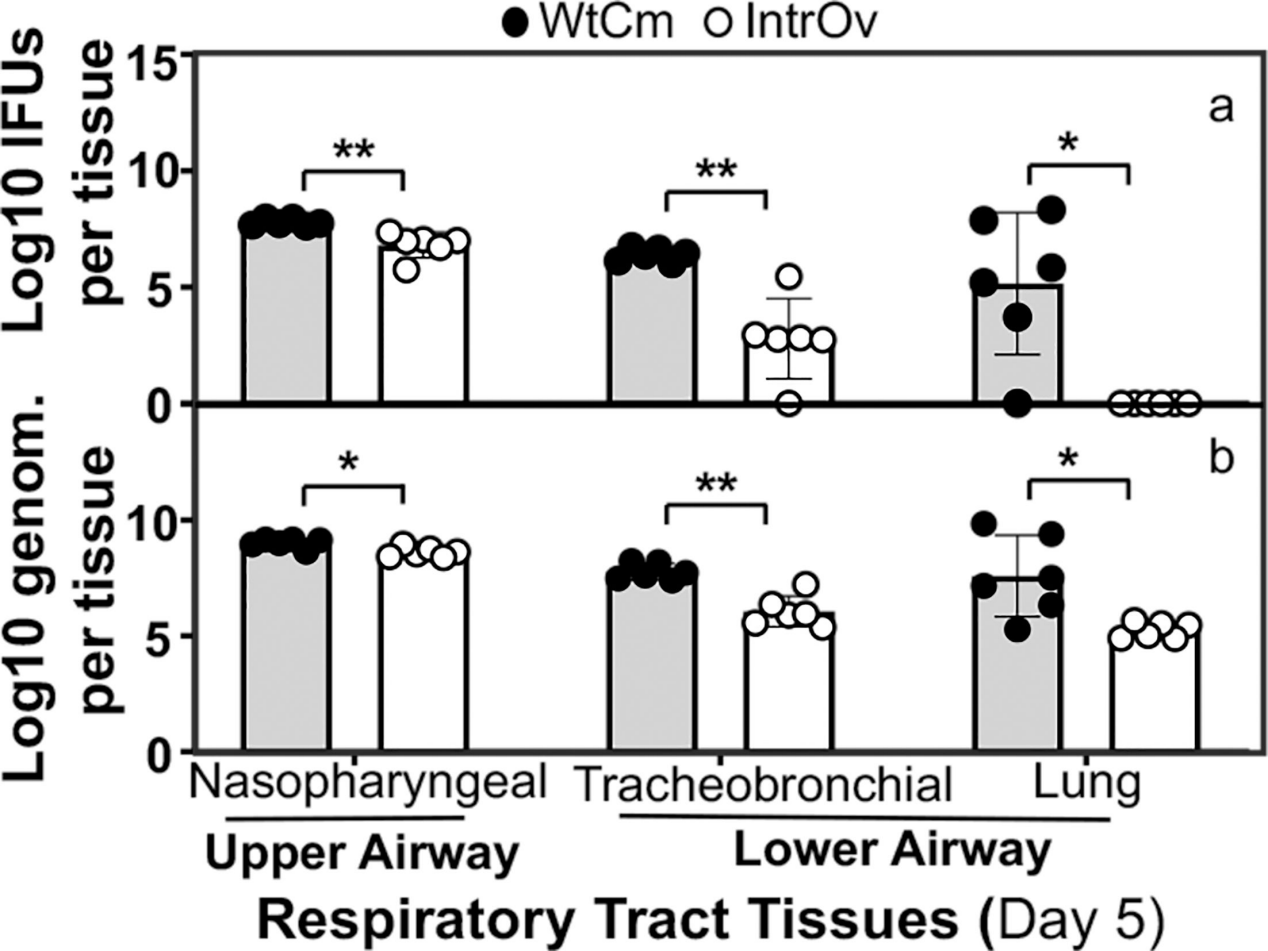
Chlamydial burdens in different regions of the respiratory tract on day 5 following intranasal inoculation. Groups of C57BL/6J mice (*n* = 6/group) were intranasally inoculated with 2 × 10⁵ IFUs of WtCm (solid circle) or intrOv (open circle). Respiratory tract tissues, including the upper airway tissues, nasal turbinates, and pharyngeal region (nasopharyngeal), and the lower airway tissues, tracheobronchial tissue, and lung tissue, as indicated along the X-axis, were harvested on day 5 post-inoculation for titrating live chlamydial organisms expressed as log10 IFUs (**a**) or chlamydial genome copies expressed as log10 genomes per tissue (**b**), as displayed along the Y-axis. Chlamydial infectious yields and genome copies were compared between the Wt CM and intrOv groups using two-tailed Wilcoxon (*P < 0.05, **P < 0.01). Note that live intrOv was absent from the lung tissue, while live WtCm was abundantly detected in the lung tissue, although both intrOv and WtCm genomes were detected.

Having demonstrated that intrOv is attenuated in causing body weight loss and shedding live organisms following intranasal inoculation, we further compared the tissue distribution of intranasally inoculated WtCm versus intrOv along the respiratory tract. We will focus only on the middle inoculum dose of 2 × 10^5^ IFUs from now on, since, at this dose, intrOv still maintained minimal toxicity while WtCm caused significant toxicity. When the loads of live chlamydial organisms harvested from the nasopharyngeal, tracheobronchial, and lung tissues 5 days following intranasal inoculation were titrated, significant levels of WtCm live organisms were detected in all three airway segments (Fig. 4A). However, intrOv live organisms were predominantly detected in the nasopharyngeal segment, with a significantly lower level in the tracheobronchial segment compared with the recovered WtCm. Surprisingly, no live intrOv was ever recovered from the lung tissues. Since the detection was performed 5 days after intranasal inoculation, when WtCm organisms are known to reach peak replication in the lung, the absence of live intrOv organisms in the lung tissue suggests that intrOv is prevented from descending to and/or replicating in the lung. Interestingly, when we simultaneously monitored chlamydial genomes in respiratory tract tissues (Fig. 4B), we found a substantial number of both WtCm and intrOv genomes across all airway tissues, although intrOv genomes were significantly fewer than WtCm genomes. This observation suggests that intrOv particles can reach lung tissue either as dead organisms or as live organisms, but the pulmonary live intrOv is either rapidly killed in the lung or prevented from developing into infectious elementary bodies (EBs).

### IntrOv-induced lung inflammation is cleared with minimal sequelae

**Fig 5:**
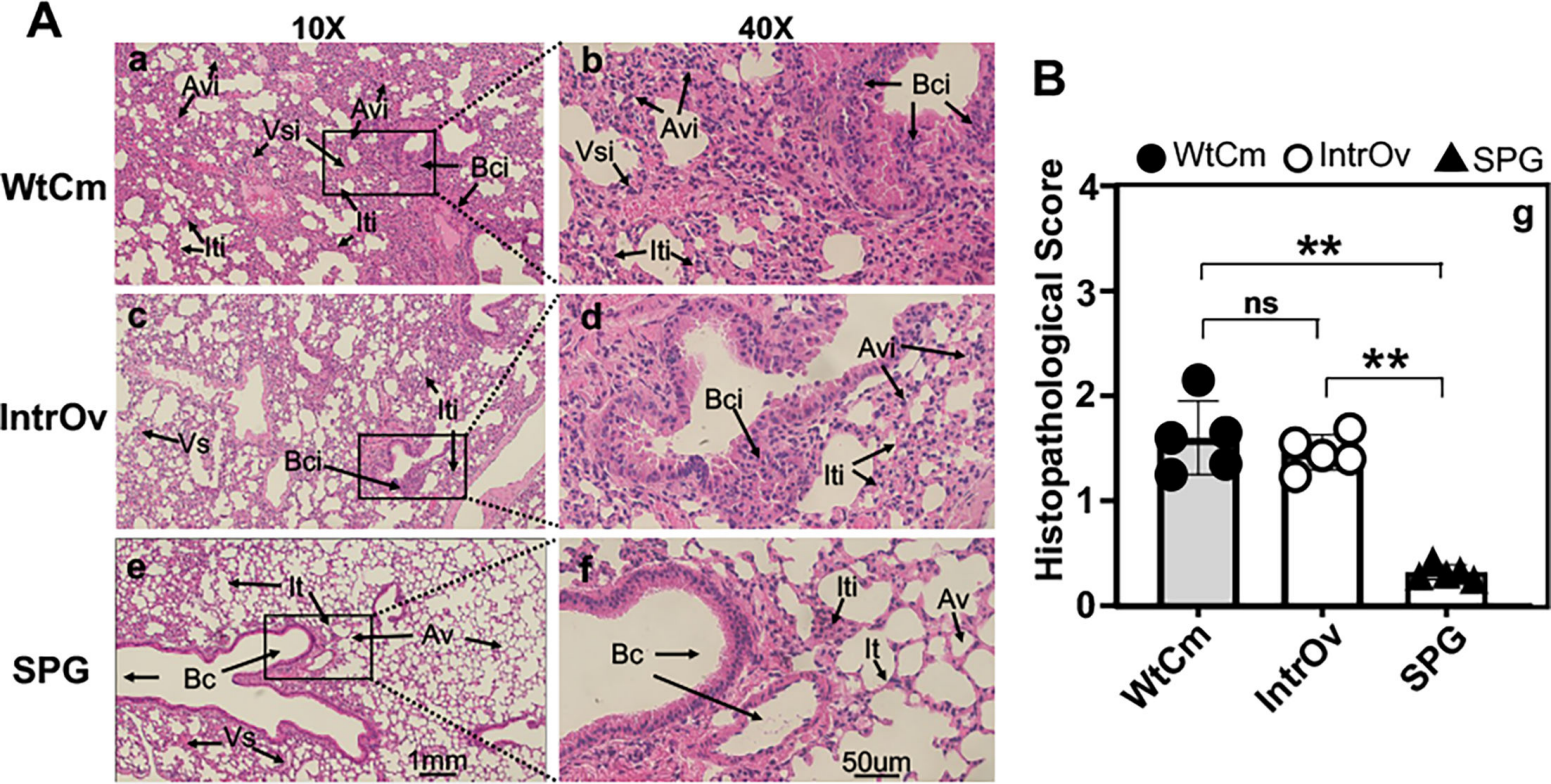
Histopathological evaluation of acute lung inflammation on day 5 following intranasal inoculation. Groups of C57BL/6J mice (*n* = 5/group) were intranasally inoculated with 2 × 10⁵ IFUs of WtCm, intrOv, or SPG buffer alone. On day 5, lung tissues were collected and processed for hematoxylin and eosin (H&E) staining. The H&E-stained lung sections were evaluated histopathologically for acute lung inflammation. (**A**) Representative H&E-stained lung sections from each group (as indicated on the left) are shown at 10× (panels a, c, and e) and 40× (panels B, d, and f), respectively. The 40× images were taken from the boxed areas of the corresponding 10× images. The branchial lumen (Bc), vascular structure (Vs), interstitial tissue (It), and alveolar structure (Av) were indicated in panels e, f, or c, while the peribranchial inflammatory infiltration (Bci), perivascular infiltration (Vsi), interstitial infiltration (Iti), and alveolar infiltration (Avi) were indicated in panels a, b, c, d, or f. (**B**) The semi-quantitative scores of overall lung histopathology evaluated and calculated as described in the Materials and Methods section were summarized and displayed along the Y-axis of panel g and compared between groups as indicated along the X-axis using two-tailed Wilcoxon. **P < 0.01; ns, not significant. Note that both WtCm and intrOv induced significant acute lung inflammation.

The lack of live intrOv, despite the presence of intrOv genomes in lung tissues following intranasal inoculation, has motivated us to evaluate histopathological inflammation in the lung. The lung tissue sections collected from mice intranasally inoculated with WtCm, intrOv, or SPG buffer alone for 5 days were evaluated for inflammatory pathologies under microscopy (Fig. 5). The overall inflammatory pathology scores were semi-quantitatively counted and calculated from the following four aspects, including peribronchial, perivascular, interstitial, and alveolar infiltration, as described in the Materials and Methods. Normal bronchial, vascular, interstitial, and alveolar structures were clearly visible in lung sections of SPG buffer alone-treated mice, although a minimal level of interstitial infiltration was also occasionally detected. In lung sections from WtCm-infected mice, extensive peribronchial, perivascular, interstitial, and alveolar infiltrates were detected, as expected. However, to our surprise, significant levels of lung inflammatory infiltrates were also detected in the intrOv-inoculated mice. The overall histopathological scores from mice infected with either WtCm or intrOv were significantly higher than those of the SPG mice, but there was no significant difference in the histopathological scores between WtCm and intrOv-infected mice.

**Fig 6:**
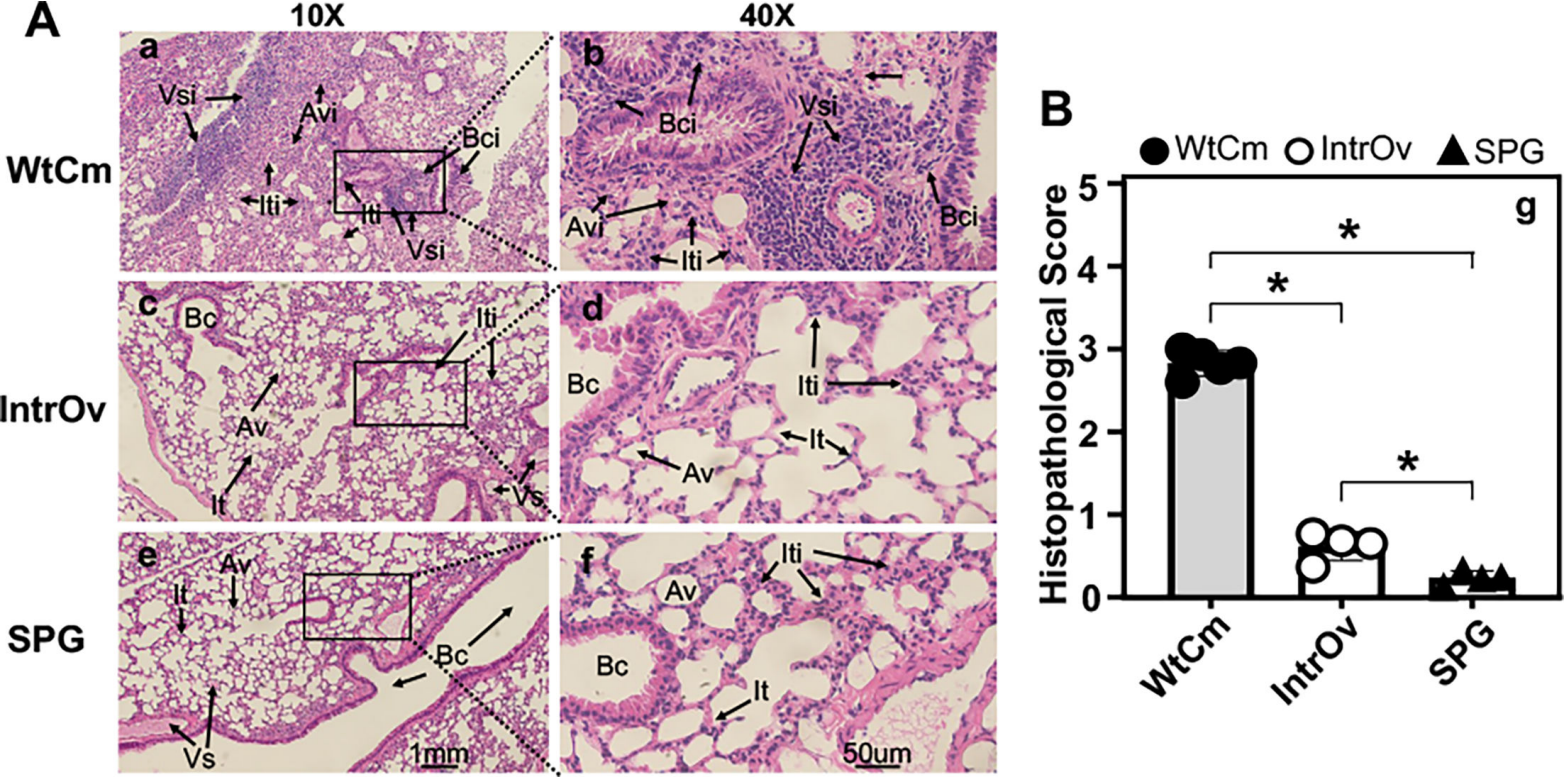
Histopathological evaluation of chronic lung inflammation on day 28 following intranasal inoculation. Groups of C57BL/6J mice intranasally inoculated with 2 × 10⁵ IFUs of WtCm, intrOv, or SPG buffer alone (*n* = 5 for WtCm, *n* = 4 for intrOv and SPG, respectively) were sacrificed on day 28 to evaluate chronic lung inflammation after H&E staining. (**A**) Representative H&E-stained lung sections from each group (as indicated on the left) are shown at 10× (panels a, c, and e) and 40× (panels b, d, and f), respectively. The branchial lumen (Bc), vascular structure (Vs), interstitial tissue (It), and alveolar structure (Av) were indicated in panels e, f, c, or d, while the peribranchial inflammatory infiltration (Bci), perivascular infiltration (Vsi), interstitial infiltration (Iti), and alveolar infiltration (Avi) were indicated in panels a, b, c, d, or f. (**B**) The semi-quantitative scores of overall lung histopathology evaluated and calculated as described in the Materials and Methods section were summarized and displayed along the Y-axis of panel g and compared between groups as indicated along the X-axis using two-tailed Wilcoxon. *P < 0.05. Note that the intrOv-induced lung inflammation was significantly lower than that induced by WtCm.

**Fig 7:**
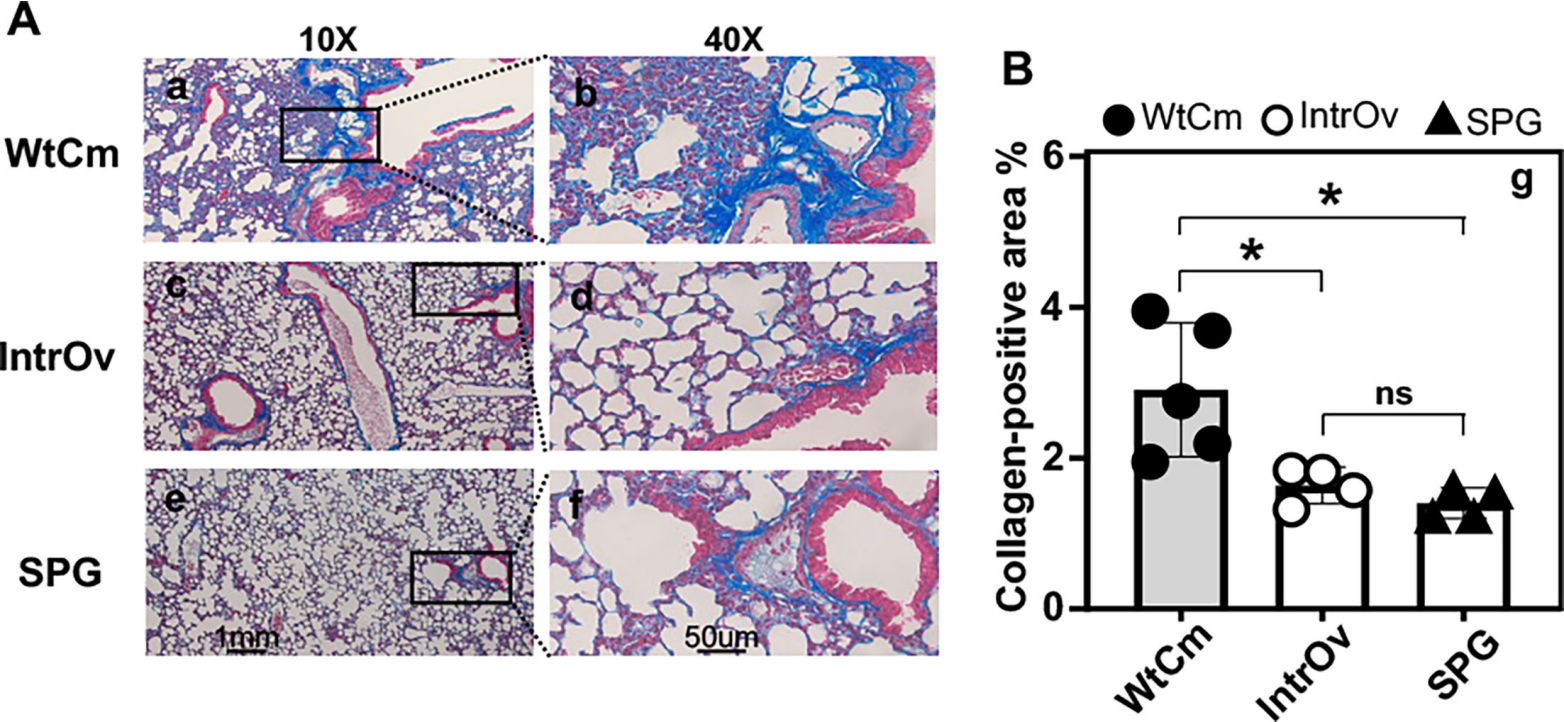
Evaluation of lung collagen deposition on day 28 following intranasal inoculation. The lung tissues harvested from the same C57BL/6J mice intranasally inoculated with 2 × 10⁵ IFUs of WtCm, intrOv, or SPG buffer alone, as described in Fig. 6 legend, were also processed for Masson’s trichrome staining to assess collagen deposition. (**A**) Representative Masson’s trichrome-stained lung sections from each group (as indicated on the left) are shown at 10× (panels a, c, and e) and 40× (panels b, d, and f), respectively. The extracellular collagen deposition or fibrosis, stained blue, was indicated with arrowheads. (**B**) The collagen-positive areas were quantified using Fiji/ImageJ, as described in the Materials and Methods section. The results, expressed as the percentage of collagen-positive area relative to the total lung tissue area, were displayed on the Y-axis of panel g and compared between groups on the X-axis using the Wilcoxon test. *P < 0.05, ns, not significant. Notably, compared with the SPG buffer control group, only WtCm, but not intrOv, induced a modest increase in collagen-positive area.

We further examined lung pathology on day 28 after intranasal inoculation (Fig. 6). We found that lung sections from WtCm-infected mice continued to show extensive peribronchial, perivascular, interstitial, and alveolar infiltrates, whereas those from SPG buffer-treated mice remained free of inflammation. In particular, the perivascular and peribronchial infiltration on day 28 in lung sections of WtCm-infected mice was even more extensive than that observed on day 5. On the contrary, the intrOv-infected mice almost completely cleared the lung inflammation initially detected on day 5. There were no significant inflammatory infiltrates in the lung sections collected from mice 28 days after intrOv inoculation. Since one of the medically relevant sequelae of airway infection is pulmonary fibrosis, we used parallel lung sections from the same mice as described above to monitor collagen deposition (Fig. 7). We found that WtCm-infected mice developed significant collagen deposition as visualized by Masson’s trichrome labeling. However, the intrOv-infected mice failed to accumulate any significant levels of collagen deposition in the lung. The % collagen-positive lung area in WtCm-infected mice was significantly higher than that from either intrOv-inoculated or SPG-treated mice. There was no significant difference in the % collagen-positive lung areas between the intrOv-inoculated and SPG buffer-treated mice.

### Intranasal inoculation with intrOv induces robust systemic IgG antibodies against chlamydial antigens

**Fig 8:**
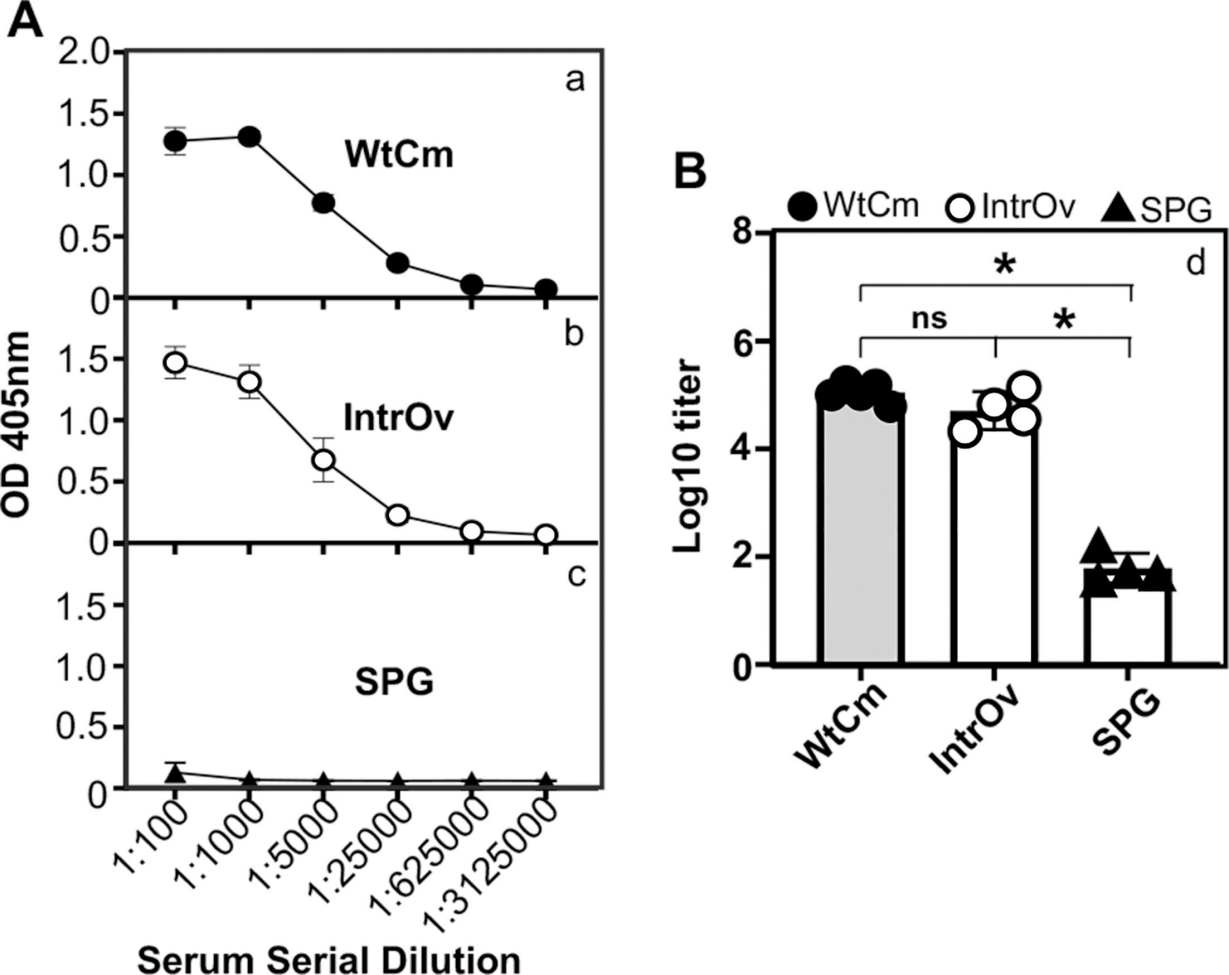
Mouse serum IgG antibody responses following intranasal inoculation. The sera were collected from the same C57BL/6J mice that were intranasally inoculated with WtCm (solid circle, panels a and d), intrOv (open circle, b and d), or SPG buffer alone (solid triangle, c and d), as described in the legend to Fig. 6. The mouse sera collected on day 28 post-inoculation were titrated for IgG antibodies against chlamydial surface antigens in an ELISA using purified WtCm elementary bodies as the coating antigen, as described in the Materials and Methods. The chlamydial antigen-specific serum IgG-binding activities measured as the optical density obtained at the wavelength of 405 nm or OD 405 nm (displayed along the Y-axis), were plotted against serial serum dilutions as indicated along the X-axis (panel **A**, a-c). The titer of the chlamydial antigen-specific IgG antibody in each serum sample determined based on OD 405 nm as described in the Materials and Methods, was converted to log10 as displayed along the Y-axis and compared between groups as indicated along the X-axis using two-tailed Wilcoxon (**B**, d). *P < 0.05; ns , not significant. Note that intranasal inoculation with IntrOv induced a robust chlamydial antigen-specific IgG antibody response as WtCm.

Since intranasal inoculation with intrOv only allowed liver organism recoveries from the upper airway and tracheobronchial segments but not from the lung tissue, we questioned whether the inoculation could still induce robust immune responses. We then used an ELISA to measure chlamydial antigen-specific antibodies in mice on day 28 post-infection (Fig. 8). The ELISA microplates were coated with gradient-purified WtCm EBs as the antigen, and a goat anti-mouse IgG as the detection antibody, allowing us to measure IgG antibodies against EB surface-exposed antigens. Unexpectedly, the intrOv-inoculated mice developed an anti-chlamydia antibody response as robust as the WtCm-infected mice. The antibody-binding activity along serum dilution curves overlapped almost completely between the intrOv- and WtCm-infected mice. The log_10_ titer in the intrOv-inoculated mice was as high as that in the WtCm-infected mice, and both were significantly higher than that in the SPG-treatment mice.

## DISCUSSION

The live-attenuated oral vaccine intrOv is being evaluated as a human vaccine against *C. trachomatis* in the genital tract (22), since oral immunization with intrOv induced transmucosal protection in the genital tract (15). For a live-attenuated vaccine, safety is a primary concern. Although intrOv has been shown to be attenuated in mucosal tissues of both the gastrointestinal (23) and genital (21, 24) tracts, it is still unclear whether intrOv is also attenuated in the airway. As WtCm is known to cause severe lung pathology and weight loss (25, 26), it is important to evaluate whether intrOv induces any airway pathology. In the current study, we first demonstrate that intrOv no longer induces significant weight loss in mice and sheds fewer live organisms than its wild-type control. Second, this reduced toxicity phenotype is consistent with the finding that intranasally inoculated intrOv is mainly restricted to the upper airway, with no live intrOv recovered from lung tissue. Third, intrOv-induced acute lung inflammation was fully resolved by day 28, whereas WtCm-induced lung inflammation persisted with progression to fibrosis. Finally, despite significant attenuation, intranasal intrOv still induced chlamydial antigen-specific serum antibodies with a titer comparable to that induced by WtCm. Thus, the current study provides the first experimental evidence of the safety of intrOv as an oral vaccine. This is because intrOv is highly attenuated in the airway, ensuring that any accidental contact of intrOv with the airway mucosa is unlikely to cause airway pathology. The combination of intrOv’s attenuation in the airway and the airway intrOv’s ability to induce systemic immune responses suggests that intrOv may also be considered a live-attenuated airway vaccine.

The mouse-adapted chlamydial species *Chlamydia muridarum* or Cm was also called the mouse pneumonitis agent (MoPn), a biowar grouped into the species of *C. trachomatis* due to Cm’s ability to cause mouse pneumonia and its initial isolation from airway-infected mouse tissue (27, 28). Mouse airway infection with Cm has been used to investigate chlamydial pathogenic mechanisms and evaluate chlamydial vaccine candidates (12). Because intranasal inoculation with Cm is known to induce body weight loss and mortality (25, 29), we first compared body weight and mortality in mice intranasally inoculated with WtCm versus intrOv each at different doses in the current study. We found that as the inoculation dose increased from low to high, WtCm induced greater body weight loss, with the peak loss occurring sooner and lasting longer. In contrast, intrOv failed to induce significant weight loss beyond the inoculation procedure when the inoculation dose was kept within the lower and middle dose ranges, suggesting that intrOv lost the ability to induce systemic toxicity at those doses. Nevertheless, intrOv still induced significant body weight loss when applied at the high inoculation dose. It is worth emphasizing that intrOv failed to induce mortality at any inoculation dose, including the highest dose of 1 × 10^8^ IFUs, although WtCm induced significant mortality even at a low dose of 1 × 10^4^ IFUs. Thus, we can conclude that intrOv is at least 10,000-fold more attenuated than WtCm, which is consistent with the finding that the intranasally inoculated intrOv shed significantly fewer live organisms than WtCm.

After comparing systemic toxicity between WtCm and intrOv across a wide range of inoculation doses from 1 × 10^4^ to 1 × 10^8^ IFUs, we focused on the lung pathology comparison at the middle dose of 1 × 10^5^ IFUs. We are aware that accidental airway contamination from the oral vaccine intrOv may involve only a small amount, which may be much lower than the 1 × 10^5^ IFU dose. The reason that we have focused on the evaluation of intrOv’s effect on the airway pathology at the dose of 1 × 10^5^ IFUs is to ensure that even if a contamination dose in the airway is as high as 1 × 10^5^ IFUs, intrOv is still safe. Furthermore, the airway safety evaluation of intrOv at the dose of 1 × 10^5^ IFUs may also provide useful information for potentially developing intrOv into a live-attenuated airway vaccine vector in the future, as intrOv at the dose of 1 × 10^5^ IFUs can induce an immune response as robust as that of the wild-type *C. muridarum*.

IntrOv does not shed any live organisms in the rectal swabs following oral inoculation and only achieves a limited shedding following intracolonic inoculation (23). However, following intravaginal inoculation, intrOv sheds as much as its WtCm control for ~4 weeks (21), although intrOv’s shedding is significantly lower than WtCm when inoculated at a low dose (24). The same study also finds that intrOv fails to efficiently ascend to the upper genital tract (24), which may partially contribute to its attenuation in pathogenicity in the upper genital tract (21). In the current study, we evaluated intrOv’s infectivity in the airway by monitoring live intrOv in oropharyngeal swabs. WtCm consistently maintains a 2-week shedding time course regardless of the inoculation dose. As the inoculation dose increases, the peak shedding time following intranasal inoculation shifts earlier. This is likely due to enhanced colonization with the higher inoculation dose. When a cultured cell monolayer is inoculated with chlamydia at a higher multiplicity of infection (MOI), multiple EBs can enter a single cell, and each EB-laden endosome fuses to form a large inclusion, or parasitophorous vacuole, where replication and production of infectious particles occur. Thus, the intracellular growth rate is accelerated at a higher MOI. Similarly, when WtCm is inoculated into the mouse airway epithelium at a higher dose, it accelerates intracellular growth and progeny production, shifting its peak shedding to the left. However, intrOv is unable to achieve a similar shift even as the inoculation dose increases, which may suggest that intrOv is not able to enter epithelial cells as efficiently as WtCm and/or the intrOv-laden endosome can’t fuse to form productive inclusions as efficiently as WtCm in the airway epithelial cells. Consistently, intrOv is significantly more dependent on centrifugation for infecting monolayer cells (24) and forms smaller plaque sizes (21) than WtCm *in vitro* cell culture systems.

Comparing with its isogenic wild-type control, intrOv is a mutant with dual mutations, a Q to E substitution mutation at the 117 position (TC0237Q117E), and a nonsense mutation in *tc0668* at 216 (TC0668G216*). Although the TC0237Q117E mutation correlates with intrOv’s growth alterations in cultured cells, whereas the TC0668G216* mutation correlates with intrOv’s attenuation in mice (21, 23, 24), the precise mechanisms remain unclear. TC0237, along with its paralogues TC0236 and TC0235, which are encoded in the same operon, is predicted to be a chlamydial outer-membrane protein with unknown function. The TC0237Q117E mutation appears to increase chlamydial invasion of epithelial cells but slow the release of intracellular infectious organisms into the extracellular environment, which may contribute to intrOv’s attenuation of infectivity in mouse mucosal tissues. TC0668 is also predicted to be a chlamydial outer-membrane protein with unknown function, although it shares structural homology with eukaryotic integrins. Because the host integrins involved have a wide range of biological activities, it is difficult to predict the function of TC0668 during chlamydial interactions with host cells. Nevertheless, TC0668 has been hypothesized to trigger host inflammatory responses that are required to drive pathological fibrosis in the genital tract (21). The same property of TC0668 may also contribute to the WtCm-induced lung fibrosis observed in the current study. Clearly, additional experiments are required to reveal the precise mechanisms by which the dual mutations contribute to intrOv attenuation in infectivity and pathogenicity. The intrOv’s failure to produce infectious chlamydial organisms in the lung and to induce pathological sequelae following intranasal inoculation may provide convenient models for investigating the corresponding mechanisms.

IntrOv is cleared from the gastrointestinal tract by IFNγ^+^ILC3s (30–32). Following an intracolonic inoculation, both WtCm and intrOv induce the intestinal IFNγ^+^ILC3s, but only WtCm is able to evade the effector mechanisms of IFNγ^+^ILC3s. The question is whether intrOv can also induce IFNγ ILC3ss or other IFNγ-producers in the airway and whether intrOv’s failure to produce infectious progenies in the lung is due to the IFNγ-producer-mediated inhibition of intrOv in the upper and/or lower airway. Experiments using the lung intrOv’s infectiousness as a readout to identify the host airway responses that prevent intrOv from producing infectious organisms in the lung are ongoing. These studies may reveal novel mechanisms for regulating airway descending infection.

Since intrOv is highly attenuated in the airway, any accidental contact of intrOv with the airway mucosa is unlikely to cause airway pathology, which further ensures the safety of intrOv as an oral vaccine. Despite attenuation, intranasal inoculation with intrOv, in the absence of any additional adjuvant, can still induce robust systemic immune responses, suggesting that intrOv may also be developed into an airway vaccine.

## MATERIALS AND METHODS

### *Chlamydia* organisms

The wild-type *Chlamydia muridarum* (WtCm) clone G13.32.1 and its isogenic mutant clone G28.51.1 were used in the current study (20, 21). The mutant clone G28.51.1 is being evaluated as an *intr*acellular oral vaccine *v*ector or intrOv due to its attenuation of genital pathogenicity and its ability to induce transmucosal protection (12). IntrOv carries dual mutations TC0237Q117E and TC0668G216*. Both intrOv and WtCm organisms were grown up in HeLa cells (human cervical carcinoma epithelial cells; ATCC# CCL-2), and density gradient centrifugation was used to purify elementary bodies (EBs). The purified EBs were stored in aliquots @ −80°C until use.

### Mouse infection

The mouse experiments were carried out in accordance with the recommendations in the Guide for the Care and Use of Laboratory Animals, endorsed by the National Institutes of Health. The protocol was approved by the Committee on the Ethics of Laboratory Animal Experiments of the University of Texas Health Science Center at San Antonio.

Seven-to-eighteen-week-old male or female mice (C57BL/6J, stock no. 000664) were purchased from Jackson Laboratories, Inc., Bar Harbor, ME. All mice were intranasally infected with intrOv or WtCm EBs at the inoculation doses of 1 × 10^4^ (low dose), 2 × 10^5^ (middle), or 1 × 10^8^ (high) inclusion-forming units (IFUs) per mouse, as indicated in individual experiments. Briefly, EBs diluted in 10 μl SPG (220 mM sucrose, 12.5 mM phosphate, 4 mM l-glutamic acid, pH 7.5) buffer were delivered to one nostril of each mouse using a p20 micropipette after isoflurane inhalation anesthetization. The mouse was held with its head up to allow it to inhale the inoculum. After inoculation, mice were monitored for body weight and/or live-organism shedding in oropharyngeal swabs, or were sacrificed to titrate live chlamydial organisms or chlamydial genome copies in respiratory tract tissues, or to measure antibodies in serum. Mouse survival was monitored daily, and survival probabilities were plotted over time using Kaplan–Meier survival curves in Prism (https://www.graphpad.com/features).

### Titrating live chlamydial organisms from mouse swabs and tissues

To monitor live chlamydial organisms shedding from the airway, oropharyngeal swabs were collected in 0.5 ml of SPG buffer and vortexed with glass beads to release infectious EBs for quantitation. For titrating live organisms from mouse respiratory tract tissues, the upper airway nasopharyngeal tissue, including the nasal turbinates and pharyngeal region, and the lower airway tissues, the tracheobronchial portion that is outside of the lung, and the lung tissue, were collected from mice sacrificed on different days after the intranasal inoculation as indicated in individual experiments. Nasopharyngeal tissues were placed in 3 ml SPG buffer, whereas tracheobronchial tissues and lung tissues were placed in 500 µL and 2 mL SPG buffer, respectively, then homogenized and sonicated. Live organisms in the supernatants were titrated on HeLa cells in duplicate. The total number of IFUs/swab or tissue was converted into log_10_ for calculating the group mean and standard deviation. Please note that the titration method’s detection limits are 10 IFUs per swab and 40 IFUs per tissue sample. For the titration of live chlamydial organisms, 150 µL of each oropharyngeal swab sample or 30 µL of each tissue homogenate sample, with serial dilutions, was used to inoculate monolayer HeLa cells. After incubation, the inoculated culture wells were processed for immunofluorescence labeling, and the chlamydial inclusions were counted to calculate the total number of live organisms recovered from each sample.

### Immunofluorescence assay

The immunofluorescence assay for visualizing and counting chlamydial inclusions in the *Chlamydia*-infected HeLa culture was described previously (33). Briefly, infected HeLa cells grown on 96-well plates were fixed with paraformaldehyde (Sigma, St. Louis, MO 63178) and permeabilized with 0.1% Triton X-100 (Thermo Scientific, A16046.0F). The monolayers were labeled with a rabbit anti-chlamydial antibody (raised by immunization with *C. muridarum* EBs) and a goat anti-rabbit IgG conjugated with fluorescein isothiocyanate (FITC, green, Jackson ImmunoResearch Laboratories, Inc.) to visualize chlamydial inclusions, while a Hoechst dye (blue; Sigma) was used for labeling nuclear DNA. The labeled cell samples were viewed under an Olympus IX-80 fluorescence microscope equipped with filter sets (Olympus, Melville, NY).

### Histopathology

Lung tissues were collected from mice at the indicated time points following intranasal inoculation and fixed in 10% neutral-buffered formalin. Fixed tissues were processed, paraffin-embedded, and sectioned at 4 μm. For histopathological evaluation, lung tissues from each mouse were sectioned into three non-consecutive sections, with adjacent sections separated by approximately 20 μm. Paraffin sections were deparaffinized, rehydrated, and subjected to hematoxylin and eosin (H&E) staining. For each section, three non-overlapping microscopic fields were randomly selected and analyzed. Inflammatory pathological changes were semi-quantitatively scored on a 0–3 scale based on the overall extent and severity of inflammatory cell infiltration in each compartment, including the peribronchial, perivascular, interstitial, and alveolar regions. Scores from all fields and sections were averaged to generate a representative histopathological score for each mouse. For assessment of collagen deposition and fibrotic changes, parallel lung sections were subjected to Masson’s trichrome staining. Collagen deposition was quantified using Fiji/ImageJ (https://imagej.net/software/fiji/) and expressed as the percentage of collagen-stained area relative to the total lung tissue area. All histopathological evaluations and image-based quantitative analyses were performed blinded, and the mean value from each mouse was used for statistical analysis.

### Enzyme-linked immunosorbent assay (ELISA)

At day 28 after intranasal inoculation, mouse sera were collected, and *Chlamydia*-specific IgG antibody responses were measured by enzyme-linked immunosorbent assay (ELISA). ELISA plates were coated overnight at 4°C with gradient-purified wild-type *Chlamydia muridarum* elementary bodies (EBs) diluted in carbonate–bicarbonate buffer (pH 9.5). After blocking and washing, serially diluted serum samples were added and incubated at 37°C for 1 h. Bound antibodies were detected using horseradish peroxidase (HRP)-conjugated goat anti-mouse IgG (cat# 1033-05, SouthernBiotech), followed by color development with an ABTS substrate (A1888, Sigma). Absorbance was measured at 405 nm using a microplate reader. Antibody responses were expressed as optical density (OD) at different serum dilutions, and endpoint titers were determined using a predefined cutoff and converted to log₁₀ for statistical analyses.

### Statistics

The number of live organisms in IFUs or genome copies at individual data points or over a time course was compared using the Wilcoxon rank-sum test. Area under the curve (AUC) was used to compare the time course or clusters of tissue sample data. When multiple groups were included in an experiment, ANOVA was first used to determine whether there was a significant overall difference among the groups. Only when *P* < 0.05 (ANOVA) were the differences between pairs of groups further analyzed using the Wilcoxon test. For qualitative data, the two-tailed Fisher’s exact test was used. Statistical significance between groups was determined using the log-rank (Mantel–Cox) test.
